# Autologous rib grafts for craniocervical junction surgery in children: a clinical application

**DOI:** 10.1186/s12891-024-07607-7

**Published:** 2024-06-26

**Authors:** Chenfu Deng, Xiaobao Zou, Haozhi Yang, Suochao Fu, Junlin Chen, Rencai Ma, Hong Xia, Xiangyang Ma

**Affiliations:** 1 Department of Spinal Surgery, General Hospital of Southern Theater Command, 111 Liuhua Rd, Guangzhou, China; 2 Southern Medical University, 1838 North Guangzhou Ave, Guangzhou, China

**Keywords:** Autologous rib grafts, Craniocervical junction, Fusion rate, Complications

## Abstract

**Objective:**

Autologous iliac bone is commonly used as a bone graft material to achieve solid fusion in craniocervical junction (CVJ) surgery. However, the developing iliac bone of children is less than ideal as a bone graft material. The matured rib bone of children presents a potential substitute material for iliac bone. The aim of this study was to evaluate the efficacy of autologous rib grafts for craniocervical junction surgery in children.

**Methods:**

The outcomes of 10 children with abnormalities of the craniocervical junction who underwent craniocervical junction surgery between January 2020 and December 2022 were retrospectively reviewed. All patients underwent posterior fusion and internal fixation surgery with autologous rib grafts. Pre- and post-operative images were obtained and clinical follow-ups were conducted to evaluate neurological function, pain level, donor site complications, and bone fusion rates.

**Results:**

All surgeries were successful. During the 8- to 24-month follow-up period, all patients achieved satisfactory clinical results. Computed tomography at 3–6 months confirmed successful bone fusion and regeneration of the rib defect in all patients with no neurological or donor site complications.

**Conclusion:**

Autologous rib bone is a safe and effective material for bone grafting in craniocervical junction fusion surgery for children that can reduce the risks of donor site complications and increase the amount of bone graft, thereby achieving a higher bone fusion rate.

## Introduction

The craniocervical junction (CVJ) is an anatomically complex transitional area between the skull and cervical spine that consists of a composite of irregular bones, ligaments, and adjacent structures, such as the vertebral artery [1]. Due to the irregularity of the bone structures, surgical treatment of the CVJ is especially difficult.

Common causes of abnormalities of the CVJ in children include trauma, infection, tumor formation, and congenital malformations leading to upper cervical instability [2, 3]. When conservative treatments are ineffective, stability can be achieved through atlantoaxial fusion (AAF) or occipitocervical fusion (OCF) [3–7]. The goal of surgical treatment for abnormalities of the CVJ in children typically involves relieving nerve compression, providing firm internal fixation, and promoting bone fusion ^[8]^. Solid internal fixation is key to promote bone fusion. The Goel–Harm technique is widely used for atlantoaxial fixation and a C1–C2 screw-rod system can provide reliable stability [5, 8–10]. However, rigid internal fixation systems only offer short-term stability, as long-term surgical outcomes are dependent on accurate bone fusion [11, 12].

Bone graft materials commonly used for posterior cervical surgery include autologous iliac bone and allograft bone. The use of autologous iliac bone can achieve a high fusion rate due to good tissue compatibility and, thus, is the gold standard for upper cervical autografts. However, complications associated with donor site tissue, such as infection and pain, remain problematic [13]. Moreover, autologous iliac bone of pediatric patients, particularly younger patients, is thin and still developing, which can result in inadequate bone grafts and negatively impact development of the iliac bone [13, 14].

The rib bones of children provide not only good structural support, but also a rich source of bone morphogenetic protein. In addition, due to excellent osteogenic properties, rib bones regenerate in most patients within 3 months after bone harvesting surgery. As compared to autologous iliac bone grafting, rib bone grafting can significantly reduce the occurrence of complications at the donor site [14]. Therefore, we attempted to use autologous rib bone as an alternative bone graft material for posterior cervical fusion surgery at the CVJ in children. Here, the clinical data of 10 pediatric patients were retrospectively analyzed to evaluate the clinical efficacy of autologous rib structural bone grafts.

## Materials and methods

The cohort of this retrospective study included 10 pediatric patients (4 males and 6 females; mean age, 6.3 ± 3.1 years; age range, 2–13 years) who underwent AAF (*n* = 7) or OCF (*n* = 3) at General Hospital of Southern Theater Command from January 2019 to December 2022. Among the 10 patients, five were diagnosed with atlantoaxial dislocation, one with os odontoideum and atlantoaxial dislocation, two with Klippel–Feil syndrome (KFS), one with os odontoideum and atlantoaxial dislocation combined with occipito-cervical fusion, and one with occipito-cervical fusion combined with KFS. All 10 patients received autologous rib structural bone grafts.

**Fig. 1:**
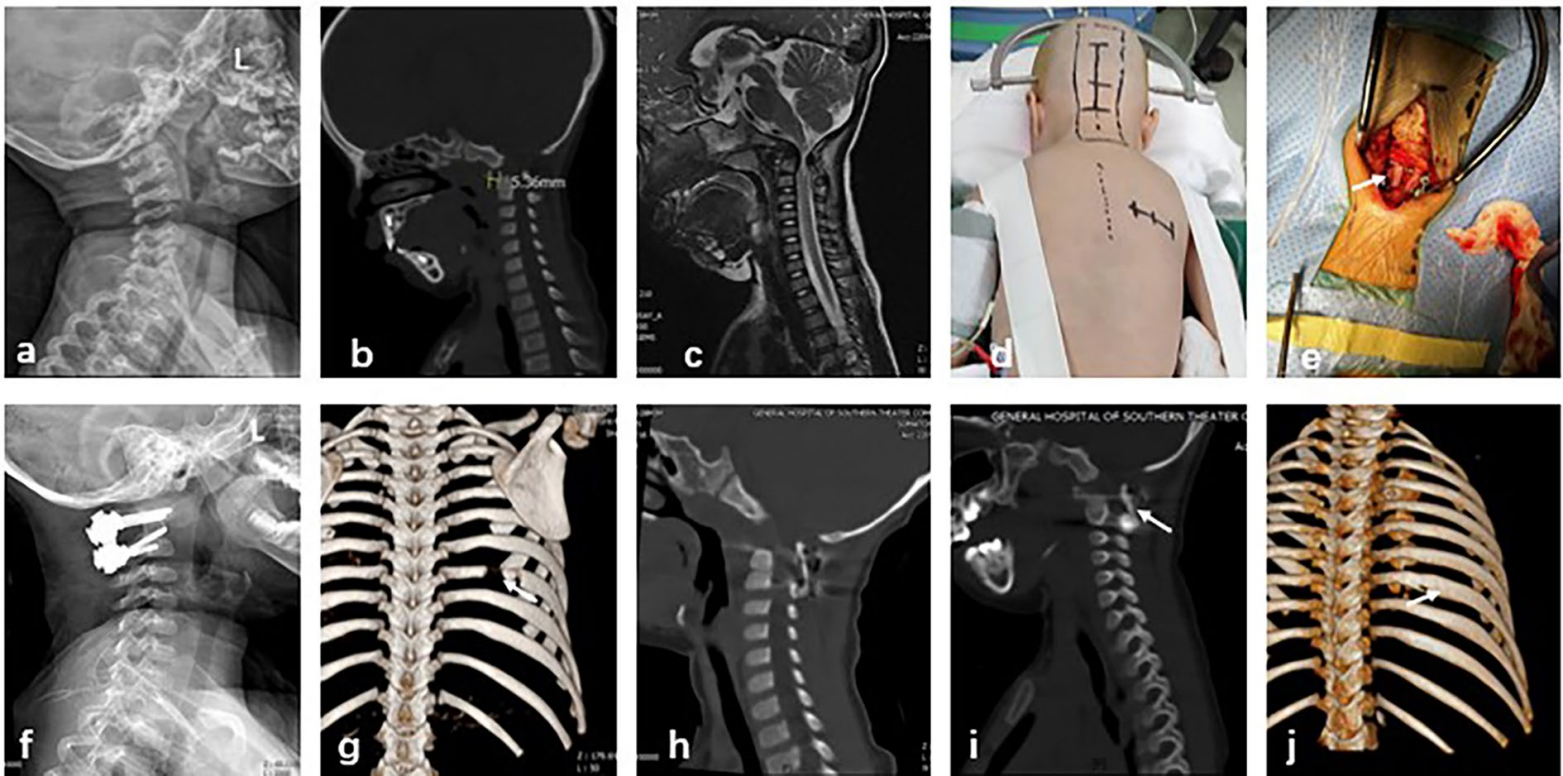
A 2-year-old female diagnosed with atlantoaxial dislocation. **a**–**c**: Preoperative X-ray, CT, and magnetic resonance imaging showing atlantoaxial dislocation, widening of the ADI, and spinal canal stenosis. **d**: Marking of the cervical incision site and oblique rib incision on the right side. **e**: Intraoperative use of autologous rib for structural bone grafting. **f**: Postoperative X-ray showing good positioning of the C1–C2 internal fixation. **g**: Chest CT 3D reconstruction at 1 week after surgery showing a defect in the right eighth rib. **h**: Sagittal CT of the cervical spine at 1 week after surgery showing good positioning of the rib bone graft. **i**: Follow-up cervical spine CT at 3 months after surgery showing bone fusion at the graft site. **j**: Chest CT 3D reconstruction at 3 months after surgery showing complete regeneration of the rib defect at the donor site

**Fig. 2:**
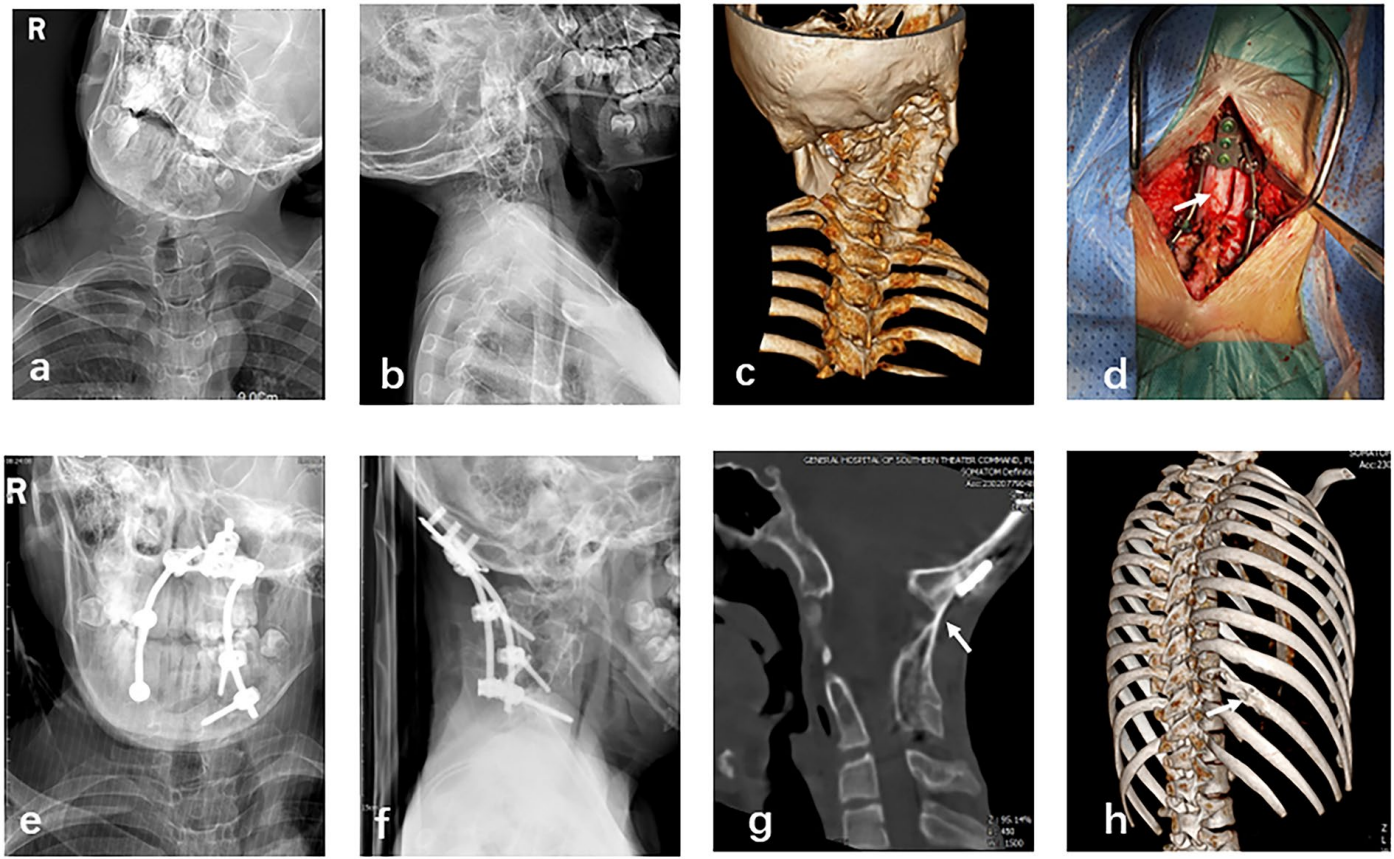
A 13-year-old male diagnosed with KFS. **a**–**c**: Preoperative X-ray and CT 3D reconstruction showing cervical deformities and partial vertebral bone fusion. **d**: Intraoperative application of C0–C6 internal fixation and autologous rib bone grafting. **e**, **f**: Postoperative X-rays of the cervical spine (sagittal and lateral views) showing good positioning of the internal fixation. **g**: Follow-up cervical spine CT at 6 months after surgery showing good bone fusion at the graft site. **h**: Chest CT 3D reconstruction at 6 months after surgery showing partial regeneration of the rib defect at the donor site

**Fig. 3:**
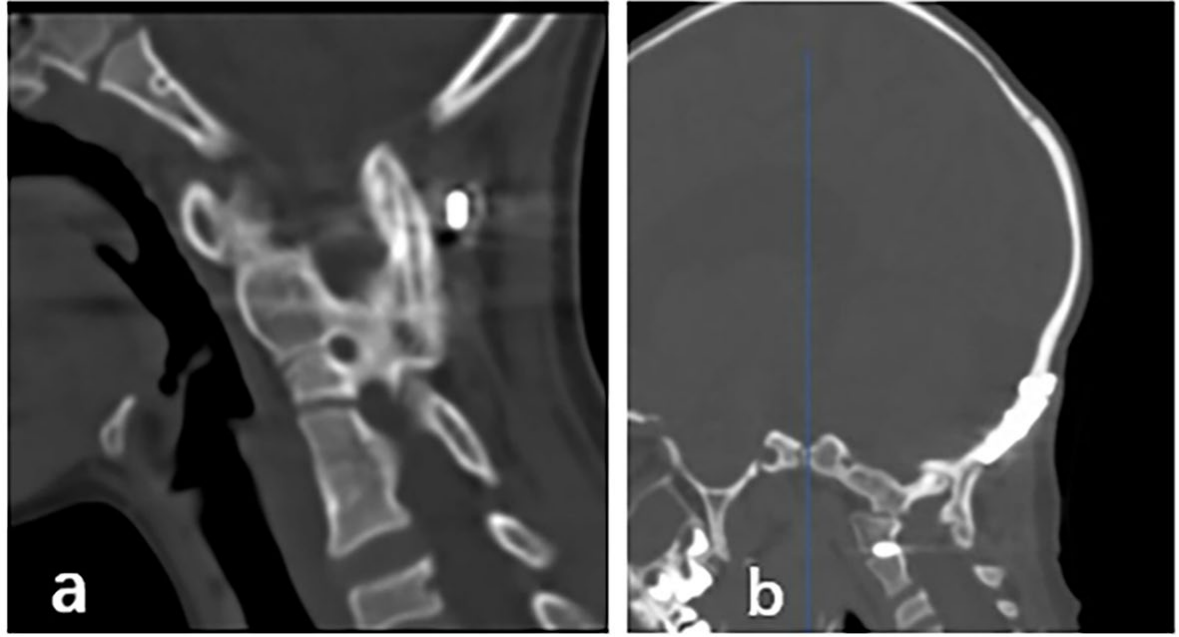
**a**, **b**: CT images of 2 children with fusion in the craniocervical junction area at 12 months and 18 months postoperatively, respectively, showed solid implant fusion in the region of the graft

Radiographic parameters, including the atlantodental interval (ADI), were evaluated by computed tomography (CT) of the cervical spine before surgery (Figs. 1a, b and c and 2a, b and c), 1 week after surgery, and at the final follow-up visit. Neurological function was evaluated with the Japanese Orthopaedic Association scoring system and the visual analogue scale (VAS). In addition, rib regeneration was assessed by chest CT three-dimensional (3D) reconstruction at 3 and 6 months after surgery. Cervical spine CT was performed at 3, 6, and 12 months after surgery until bone graft fusion was complete (Fig. 3a and b), which was defined as the presence of continuous bone trabeculae on sagittal CT images in the bone graft area.

### Surgical technique

All patients underwent routine skull traction in the prone position and the surgical site was selected by fluoroscopy. Incisions were made along the posterior midline and the dorsal oblique rib (Fig. 1d). An appropriate length of bone was harvested from the middle segment of the 8th to 10th rib (Fig. 1g) using the periosteal detachment technique to avoid injury to the intercostal nerves and vessels. After placement of the internal fixation system, a bone graft bed was prepared in the C0–C2 or C1–C2 region. Then, the cortical bone of the posterior arch and spinous process of the atlas and axis were removed using a high-speed bur and an appropriately positioned slot was prepared. The rib was trimmed to an appropriate length and split longitudinally along the center to increase the contact surface of cancellous bone. The prepared rib was then placed in the prepared slot between the atlas and axis or C0–C2 region as a structural bone graft (Figs. 1e and 2d), while the remaining rib was crushed and spread around the rib. There is a slight difference in the method of applying rib implants in the atlantoaxial and occipitocervical regions: in the atlantoaxial region the implant bed is prepared from the posterior arch of the atlas to the spinous process of the pivot vertebrae, whereas in patients with occipitocervical fusion the implant is placed in the area of the occipital plate beneath the occipital bone between the spinous process of the pivot vertebrae and the vertebral plate. In both cases, the implant bed is prepared by grinding the cortical bone of the implant bed with a high-speed grinder and slotting the implant in the appropriate location for placement of the rib implant. Occipitocervical fusion requires more bone volume than atlantoaxial fusion, and the rib preparation requires the removal of more ribs and, if necessary, the cutting of two adjacent ribs for the implant material. A drainage tube was placed and the incision was closed layer by layer.

### Postoperative management and follow-up

All patients received intravenous cephalosporin antibiotics within 24 h after surgery to prevent infection. On postoperative day 3, the drainage tube was removed if the drainage volume was < 30 mL and the patient was allowed to ambulate with the use of a cervical collar, which was continued for an average of 3 months.

Clinical outcomes were assessed by radiographic evaluation, including X-ray and CT scans (Figs. 1f and h and 2e and f), in addition to ADI, VAS, and JOA scores within 1 week after surgery. Imaging parameters were reviewed at 3, 6, 9, and 12 months after surgery and then every 12 months during outpatient follow-up visits. Chest CT 3D reconstruction was performed at 3 and 6 months postoperatively to assess rib regeneration.

### Statistical analysis

Statistical analysis was performed using IBM SPSS Statistics for Windows, version 25.0. (IBM Corporation, Armonk, NY, USA). The paired sample *t*-test was used to compare preoperative and final ADI, JOA, and VAS scores. A probability (*p*) value < 0.05 was considered statistically significant.

## Results

The mean operation time was 167.1 ± 37.0 min, the average intraoperative blood loss was 102 ± 79.7 mL, the mean bone fusion time after surgery was 3.3 ± 0.9 months, and the mean follow-up time was 13.8 ± 4.8 months (Table 1). Neurological symptoms were improved in all patients after surgery and there were no complications of vertebral artery injury or cerebrospinal fluid leakage. Bone fusion at the graft site was observed in all patients at 3–6 months after surgery (Figs. 1i and 2g). There were no complications at the rib donor site. All patients exhibited good rib regeneration at the donor site on chest CT 3D reconstruction (Figs. 1j and 2h) at 3–6 months after surgery (Table 2).

**Table 1:** Patient information

Case	Sex/age	Diagnosis	Fusion level	Blood loss volume (mL)	Operation time (min)
1	F/6	KFS	C0–C2	100	217
2	M/13	KFS	C0–C4	300	240
3	F/6	AD, OO	C1–C2	50	130
4	M/8	AAD	C1–C2	100	133
5	M/4	AD, OO, AA	C1–C2	100	150
6	F/2	AAD	C1–C2	150	186
7	F/4	AAD	C1–C2	30	145
8	M/5	AAD	C1–C2	20	145
9	F/9	AAD	C1–C2	70	175
10	F/6	AA, KFS	C0–C2	100	150
Mean ± SD				167.1 ± 37.0	102 ± 79.7

Abbreviations: AA, assimilation of atlas; AAD, atlantoaxial dislocation; F, female; KFS, Klippel–Feil syndrome; M, male; OO, os odontoideum; SD, standard deviation

**Table 2:** Preoperative and final imaging and neurofunctional indicators

Case	preADI score	postADI score	preVAS score	postVAS score	preJOA score	postJOA score	Fusion time confirmed (months)	Follow-up (months)	Complication
1	3.23	2.25	2	0	14	16	3	12	no
2	2.52	1.64	3	0	14	16	3	8	no
3	10.43	1.17	4	2	12	15	3	14	no
4	6.45	2.15	3	1	14	17	6	16	no
5	5.72	1.14	3	1	14	16	3	18	no
6	5.36	1.28	3	0	14	17	3	8	no
7	7.56	1.57	3	0	13	16	3	24	no
8	5.16	1.67	3	0	15	17	3	12	no
9	8.83	2.17	4	1	14	17	3	12	no
10	5.45	1.53	3	0	14	16	3	14	no
Mean ± SD	6.07 ± 2.39	1.65 ± 0.41	3.10 ± 0.67	0.50 ± 0.71	13.80 ± 0.79	16.30 ± 0.67	3.3 ± 0.95	13.80 ± 0.76	
t	5.57		15.92		−15.00				
*p*	0.000		0.000		0.000				

Abbreviations: preADI, preoperative ADI; *p*, probability; preJOA, preoperative JOA; preVAS, preoperative VAS; postADI, postoperative ADI; postJOA. postoperative JOA; postVAS, postoperative VAS; SD, standard deviation; t, t-statistic

## Discussion

Surgical treatment for abnormalities of the CVJ in children is especially difficult for spinal surgeons. A screw-rod internal fixation system is often applied for craniocervical junction surgery in children to achieve stability of the reconstructed CVJ [5, 15, 16]. Firm internal fixation is key to successful bone fusion. However, loosening of the internal fixation will likely lead to non-union of the bone graft, requiring revision surgery and causing greater damage to the patient [12, 17]. Hence, complete fixation and fusion are particularly important to achieve good outcomes from spinal fusion surgery. With the development of spine surgery, autologous bone is a common source for bone grafts due to good tissue compatibility [18].

In adults, autologous iliac bone grafting is the gold standard for craniocervical junction fusion [19, 20]. However, there are obvious limitations to autologous iliac bone grafting in pediatric patients, such as unavoidable donor site complications, long surgical durations, increased blood loss, and greater risks of infection [21]. Notably, some patients experience long-term intractable postoperative neuropathic pain after iliac bone grafting and many develop complications, especially infection. Moreover, the iliac bone is not fully developed in pediatric patients, thus harvesting the iliac bone may adversely impact normal bone development. Second, the iliac bone of pediatric patients is thinner and softer than that of adults and cannot provide adequate bone grafting material and structural support [8]. In addition, this population has increased risks of complications of nerve pain and infection caused by iliac bone grafting. Therefore, we recommend the use of autologous rib bone as the graft material for craniocervical junction fusion surgery in children.

In an early report, Brockmeyer [19] summarized the technique of the rib structure grafting combined with internal fixation for treatment of atlantoaxial dislocation in children and found that grafting of the rib combined with transarticular screws at C1–2 achieved good stability and improved the fusion rate. As compared to autologous bone, the use of allogenous bone is limited by increased risks of immune rejection and infection, the lack of osteogenic characteristics, and increased costs to the patient. The use a combination of allogenous and autologous bone can improve the bone fusion rate, but this approach is not commonly applied in CVJ fixation surgery for pediatric patients. An ideal bone graft material should serve two important functions: promote bone formation and provide structural support [7, 22]. Therefore, some researchers have begun to explore the use of autologous rib bone as the graft material for posterior CVJ fusion surgery in children. Autologous rib grafts in children have a good cortical bone surface that can provide structural bone graft support, increase the contact stress between the bone graft and bed, and are rich in bone morphogenetic protein [14], which can significantly improve short-term fusion rates and reduce the risk of revision surgery. In addition, the good osteogenic function of the developing ribs of children [14] can promote bone regeneration within 3–6 months, thereby avoiding permanent bone defects at the donor site. Blunt dissection under the periosteum is recommended when exposing the rib to avoid damage to the intercostal nerve and subsequent postoperative neuropathic pain and to avoid damage to the pleura and potential pneumothorax [22]. Resection of the posterior atlantoaxial arch is rarely required in pediatric patients with atlantoaxial dislocation, and there were no relevant cases in this study. We believe that even if resection of the posterior atlantoaxial arch is required, there is still room for bone grafting as long as part of the posterior atlantoaxial arch is preserved. Structural bone grafting of the ribs, in which a strip of ribs is stuck on the graft bed, provides better stability than flat grafting, with a theoretically smaller risk of displacement or compression of the spinal cord. Compared with patients without posterior arch resection, we believe that partial posterior arch resection patients will probably have a lower fusion efficiency because of the reduced implant contact area, although more clinical case data are needed to support this view. In this study, bone fusion was achieved in all 10 patients at the last follow-up (fusion rate of 100%) and the rib had completely regenerated within 3–6 months after surgery with no complications at the donor site.

In summary, autologous rib grafting offers significant advantages to posterior CVJ surgery and can be widely used in pediatric patients. However, the patient cohort of this study was relatively small, thus long-term follow-up studies of larger populations are needed to confirm the long-term efficacy.

## Conclusion

The results of this study demonstrate the advantages of autologous rib bone as graft material for posterior CVJ fusion surgery for pediatric patients, which include high fusion rates and few complications. Thus, autologous rib bone should be considered as a good source of grafting material for CVJ fixation surgery for pediatric patients.

## Data Availability

The data used and analyzed during the current study are available in anonymized from the corresponding author on reasonable request.
